# Seroprevalence of cytomegalovirus in individuals on antiretroviral therapy in a Nigerian tertiary hospital

**DOI:** 10.1186/s12879-026-13138-4

**Published:** 2026-03-21

**Authors:** Baba-Ali Fatima, Ahmad Hayatu, Umar Mohammed Hassan, Kabiru Muhammad, Ahmed Yusuf

**Affiliations:** 1 Federal Teaching Hospital, Gombe, Gombe State Nigeria; 2 Usmanu Danfodiyo University, Sokoto, Sokoto state Nigeria; 3 United Lincolnshire Teaching Hospital, NHS Trust Lincoln County Hospital, Greetwell Road, Lincoln, LN2 5QY UK

**Keywords:** HIV, Cytomegalovirus, Antiretroviral therapy, Co-infection, Nigeria

## Abstract

**Background:**

Cytomegalovirus (CMV) is a ubiquitous herpesvirus with nearly universal seroprevalence in sub-Saharan Africa. In people living with HIV, CMV contributes to immune activation, systemic inflammation, and accelerated immune dysfunction despite virologic suppression on antiretroviral therapy (ART). Data on CMV–HIV co-infection remain scarce in North-East Nigeria, where fragile health systems and late presentation to care are common.

**Methods:**

We conducted a cross-sectional study among 181 adults with confirmed HIV receiving ART at the Federal Teaching Hospital, Gombe. Socio-demographic and clinical data were collected using structured questionnaires and patient records. Serum samples were tested for CMV immunoglobulin G (IgG) and immunoglobulin M (IgM) antibodies using ELISA. CD4 + T-cell counts and HIV viral load were measured using flow cytometry and real-time PCR.

**Results:**

All participants (181/181; 100%) were CMV IgG positive, confirming universal prior exposure. CMV IgM seropositivity—indicating recent or ongoing infection—was detected in 58 of 181 participants (32.1%). IgM-positive cases were distributed across all age groups and were more frequent among women. Patterns of IgM seropositivity across CD4 + T-cell and viral load categories reflected the underlying distribution of clinical characteristics in the cohort.

**Conclusion:**

This study provides the first evidence of universal CMV exposure and a substantial prevalence of recent or reactivated infection among adults with HIV on ART in North-East Nigeria. The presence of CMV IgM positivity in a clinically stable, largely virologically suppressed population highlights CMV’s persistent immunologic relevance. Further research using molecular assays is warranted to clarify the role of CMV reactivation in immune dysfunction and long-term ART outcomes.

**Clinical trial:**

Not applicable.

**Supplementary Information:**

The online version contains supplementary material available at 10.1186/s12879-026-13138-4.

## Background

Human immunodeficiency virus (HIV) remains a leading cause of global morbidity and mortality despite major advances in antiretroviral therapy (ART). Recent global estimates from UNAIDS indicate that approximately 40.8 million people were living with HIV worldwide in 2024, with about 1.3 million new infections and 630,000 AIDS-related deaths reported in the same year. Expanded access to antiretroviral therapy has resulted in approximately 31.6 million people receiving ART globally, reflecting continued progress in epidemic control, although significant regional disparities remain [1]. 

Nigeria continues to bear a substantial share of the HIV burden in West and Central Africa, with an estimated ~ 2.0 million people living with HIV and adult prevalence around 1.3–2.0%, despite gradual declines in incidence and improved ART coverage [2, 3]. These highlight ongoing transmission dynamics and reinforce the importance of monitoring opportunistic co-infections such as cytomegalovirus (CMV), particularly in resource-limited settings. While ART scale-up has significantly improved survival and reduced transmission, opportunistic infections still contribute substantially to morbidity and mortality, especially in resource-limited settings where late presentation to care is common.

Among opportunistic pathogens, human cytomegalovirus (CMV) has particular relevance. CMV is a ubiquitous β-herpesvirus with adult seroprevalence ranging from 40% to nearly 100% globally and consistently higher levels in low- and middle-income countries [4]. In people with HIV, CMV co-infection is almost universal and is associated with significant clinical outcomes including retinitis, colitis, and pneumonitis. Importantly, CMV also drives persistent immune activation and systemic inflammation, processes that can accelerate disease progression even in virally suppressed patients [5]. Evidence indicates that CMV reactivation may amplify HIV replication, expand the latent reservoir, and impair immune restoration, underscoring its role as a critical cofactor in HIV pathogenesis.

Beyond acute end-organ disease, CMV has been linked to immunosenescence. A recent study from India demonstrated that higher anti-CMV antibody levels in people with HIV on long-term ART correlated with reduced naïve T-cell proportions, expansion of late-differentiated phenotypes, and elevated soluble CD14 levels, all markers of immune aging (Shete et al., 2024) [6]. Such findings highlight how CMV may contribute to premature immune dysfunction and age-related comorbidities in populations already vulnerable to accelerated aging with HIV. In SSA, where CMV seroprevalence is near-universal, these effects may be particularly consequential for long-term ART outcomes [4]. 

Despite its potential clinical importance, CMV remains under-studied in Nigeria. Available reports are geographically limited. For example, studies in Ilorin (North-Central Nigeria) and Edo State (South-South) documented high CMV seropositivity among patients on ART, with evidence of both latent and active infection [7, 8]. These findings mirror global patterns but may not be generalizable to other regions. In the North-East, where insecurity, displacement, and fragile health systems shape access to care, no peer-reviewed studies have assessed CMV–HIV co-infection among adults in ART programs. This lack of data hampers the ability to evaluate whether CMV contributes to immune activation, unsuppressed viremia, or clinical decline in this context.

The present study was designed to address this gap by investigating the prevalence and pattern of CMV infection among adults receiving ART in Gombe State, North-East Nigeria. Specifically, we measured CMV immunoglobulin G (IgG) and immunoglobulin M (IgM) antibodies and examined their associations with CD4 + T-cell counts, HIV viral load, and treatment characteristics. By generating the first CMV–HIV epidemiologic data from this region, this study provides locally relevant evidence to guide clinical and public health decision-making. Findings may inform whether CMV testing or monitoring should be prioritized in HIV programs in resource-limited settings, where optimizing ART outcomes remains a critical priority.

## Methods

### Study design and setting

We conducted a cross-sectional study at the Antiretroviral Therapy (ART) Clinic of the Federal Teaching Hospital, Gombe (FTHG), North-East Nigeria. FTHG is a 451-bed tertiary hospital serving as a referral center for Gombe State and neighboring states in the region. The ART clinic manages more than 14,000 patients annually, reflecting both local demand and referrals from surrounding conflict-affected states.

### Study population and eligibility

Adults (≥ 18 years) with confirmed HIV infection receiving ART at FTHG were consecutively recruited. Inclusion criteria were documented HIV seropositivity, current use of ART, and provision of written informed consent. Patients who were HIV-negative, not on ART, or with other known viral infections (e.g., hepatitis B or C) were excluded.

### Sample size

The minimum sample size was estimated using Cochran’s formula, assuming a CMV prevalence of 11.3% from a prior Nigerian study [9], a 95% confidence interval, and a 5% margin of error. The calculated sample size of 154 was increased by 20% to account for attrition, giving a final target of 181 participants.

### Participant recruitment

Study participants were enrolled using a consecutive recruitment approach, in which all individuals who met the eligibility criteria and attended the ART Clinic during the study period were invited to participate. Recruitment was conducted in collaboration with the clinical team responsible for the care of people living with HIV, who assisted in identifying adults receiving antiretroviral therapy who satisfied the inclusion and exclusion criteria. Eligible individuals were approached, the study procedures were explained, and written informed consent was obtained prior to participation. Enrollment continued until the predetermined sample size of was achieved.

### Data collection

A structured interviewer-administered questionnaire developed specifically for this study was used to obtain socio-demographic and behavioral data, including age, sex, marital status, education, occupation, and family setting. Clinical information was obtained from patient records where appropriate. (see Supplementary File 1)

### Specimen collection and laboratory analysis

Approximately 10 mL of venous blood was collected from each participant. Whole blood was used immediately for CD4 + T-cell enumeration in accordance with the BD FACSCount protocol, and therefore was not stored prior to analysis. Following clotting and centrifugation, serum was separated and stored at − 20 °C for CMV serology. All serum samples were analysed within seven days, consistent with the maximum stability period recommended by the ELISA kit manufacturer. Plasma for HIV viral load testing was processed promptly after centrifugation and was not subjected to long-term storage before analysis.

CD4 + T-cell counts were determined from freshly collected whole blood using flow cytometry (BD FACSCount, Becton Dickinson, USA). HIV viral load was quantified using real-time polymerase chain reaction (PCR) on the COBAS AmpliPrep/COBAS TaqMan platform (Roche Diagnostics, Indianapolis, USA; detection range: 40–10,000,000 copies/mL). CMV IgG and IgM antibodies were measured on stored serum using a commercial enzyme-linked immunosorbent assay (ELISA) kit (Melsin, China) following the manufacturer’s instructions. IgG positivity indicated prior CMV exposure, while IgM positivity was interpreted as evidence of recent or reactivated infection.

### Statistical analysis

Data were analysed using SPSS version 21.0 (IBM Corp., Armonk, NY, USA). Continuous variables are summarised as means and standard deviations, while categorical variables are reported as frequencies and percentages. The primary objective of the study was descriptive: to determine CMV IgG and IgM seroprevalence among adults on ART and to describe the distribution of IgM-positive cases across socio-demographic and clinical categories. No inferential hypothesis testing was performed for subgroup comparisons because the study was not designed or powered to evaluate associations between CMV serostatus and clinical or demographic variables.

### Ethical considerations

The study received ethical approval from the Research and Ethics Committee of the Federal Teaching Hospital, Gombe (NHREC/25/10/2013). Written informed consent was obtained from all participants (see Supplementary File 2). The study was conducted in accordance with the principles of the Declaration of Helsinki.

## Results

### Participant characteristics

A total of 181 HIV-positive adults on ART were enrolled. The mean age was 41.2 years (SD ± 10.7, range 19–78). Most participants were female (67.4%, *n* = 122) and married (60.8%, *n* = 110). Secondary (36.5%, *n* = 66) and tertiary (40.9%, *n* = 74) education levels were the most common. With respect to occupation, the largest groups were civil servants (37.0%, *n* = 67), artisans (30.4%, *n* = 55), and business owners (26.0%, *n* = 47), while 6.6% (*n* = 12) were housewives (Table 1).

**Table 1:** Socio-demographic characteristics of study participants

Variables	Frequency	Percentage (%)
**Age Groups**		
11–20	2	1.1
21–30	19	10.5
31–40	66	36.5
41–50	58	32.0
51–60	22	12.2
61–70	11	6.1
71–80	3	1.7
**Gender**		
Male	59	32.6
Female	122	67.4
**Tribes**		
Tangale	66	36.5
Fulani	30	16.6
Hausa	21	11.6
Tera	11	6.1
Others	53	29.3
**Marital Status**		
Single	22	12.2
Married	110	60.8
Divorced	15	8.3
Widowed	34	18.8
**Education**		
Qur’anic/Arabic	10	5.5
Primary	22	12.2
Secondary	66	36.5
Tertiary	74	40.9
None	9	5.0
**Occupation**		
Civil Servants	67	37.0
Housewives	12	6.6
Business	47	26.1
Artisans	55	30.4

Table 2 summarises the distribution of CMV IgM and IgG serostatus across socio-demographic characteristics. CMV IgG was detected in all participants (181/181, 100%), confirming universal prior exposure in this cohort. CMV IgM was identified in 58 participants (32.1%), indicating recent or reactivated infection. IgM-positive cases were distributed across all age groups, with the highest proportions in the 31–40 years (22/58, 37.9%) and 41–50 years (16/58, 27.6%) categories. Women accounted for a greater proportion of IgM-positive cases than men (39/58, 67.2% vs. 19/58, 32.8%). IgM seropositivity also varied across tribes, marital status, education and occupation. These values are descriptive; subgroup percentages for CMV IgM were recalculated using the total number of IgM-positive participants (*n* = 58) as the denominator.

**Table 2:** Distribution of CMV IgM and IgG serostatus across socio-demographic characteristics

Variables	CMV IgM *n* (%)*	CMV IgG *n* (%)
**Age Groups**		
11–20	1 (1.7%)	2 (1.1%)
21–30	4 (6.9%)	19 (10.5%)
31–40	22 (37.9%)	66 (36.5%)
41–50	16 (27.6%)	58 (32.0%)
51–60	8 (13.8%)	22 (12.2%)
61–70	4 (6.9%)	11 (6.1%)
71–80	3 (5.2%)	3 (1.7%)
**Gender**		
Male	19 (32.8%)	59 (32.6%)
Female	39 (67.2%)	122 (67.4%)
**Tribes**		
Tangale	20 (34.5%)	66 (36.5%)
Fulani	8 (13.8%)	30 (16.6%)
Hausa	10 (17.2%)	21 (11.6%)
Tera	3 (5.2%)	11 (6.1%)
Others	17 (29.3%)	53 (29.3%)
**Marital Status**		
Single	10 (17.2%)	22 (12.2%)
Married	30 (51.7%)	110 (60.8%)
Divorced	5 (8.6%)	15 (8.3%)
Widowed	13 (22.4%)	34 (18.8%)
**Education**		
Qur’anic/Arabic	4 (6.9%)	10 (5.5%)
Primary	2 (3.4%)	22 (12.2%)
Secondary	23 (39.7%)	66 (36.5%)
Tertiary	25 (43.1%)	74 (40.9%)
None	4 (6.9%)	9 (5.0%)
**Occupation**		
Civil Servants	24 (41.4%)	67 (37.0%)
Housewives	4 (6.9%)	12 (6.6%)
Business	15 (25.9%)	47 (26.0%)
Artisans	15 (25.9%)	55 (30.4%)

*CMV IgM percentages calculated using *n* = 58 (total IgM-positive). CMV IgG percentages calculated using *n* = 181 (total participants)

Table 3 presents the distribution of CMV IgM and IgG serostatus according to ART-related characteristics. CMV IgG was detected in all participants (181/181, 100%). CMV IgM was present in 58 participants (32.1%). Among IgM-positive individuals, the majority were receiving first-line ART 50 (86.2%), while 8 (13.8%) were on second-line treatment. IgM positivity was also observed across all ART duration categories and was most frequent among participants on ART for more than five years (49/58, 84.5%). These values describe the distribution of IgM-positive cases across treatment groups; percentages have been recalculated using the total number of IgM-positive participants (*n* = 58). No inferential statistical testing was performed, as the study was not designed or powered to evaluate associations between ART characteristics and CMV serostatus.

**Table 3:** Distribution of CMV IgM and IgG serostatus across ART-related characteristics

Variables	CMV IgM *n* (%)*	CMV IgG *n* (%)
**Regimen of Treatment**		
1st Line	50 (86.2%)	164 (90.6%)
2nd Line	8 (13.8%)	17 (9.4%)
**Duration on ART**		
< 1 year	2 (3.4%)	7 (3.9%)
1–5 years	7 (12.1%)	24 (13.3%)
> 5 years	49 (84.5%)	150 (82.9%)

*CMV IgM percentages calculated using *n* = 58 (total IgM-positive). CMV IgG percentages calculated using *n* = 181 (total participants)

Table 4 summarises the distribution of CMV IgG and IgM across CD4 + T-cell and HIV viral load categories. CMV IgG was observed across all CD4 strata and viral load categories, reflecting widespread prior exposure. Overall CMV IgM seropositivity was observed in 58/181 participants (32.1%); IgM-positive participants were distributed across CD4 categories, with numerically higher counts in the 251–750 cells/µL range (14–17 cases in the 251–500 and 501–750 categories). IgM positivity was also present across viral load categories and was most frequently observed among participants with undetectable viral load.

**Table 4:** Distribution of CMV serostatus by CD4 + T-cell count and HIV viral load

Variables	CMV IgM *n* (%)	CMV IgG *n* (%)
**CD4 + Count (cells/µL)**		
1–250	5 (3.6)	14 (10.2)
251–500	14 (10.2)	59 (43.1)
501–750	17 (12.4)	41 (29.9)
751–1000	6 (4.4)	14 (10.2)
> 1000	5 (3.6)	9 (6.6)
**HIV Viral Load** **(copies/mL)**		
Target Not Detected	35 (25.9)	111 (82.2)
< 20	0(0)	0 (0)
21–400	7 (5.2)	18 (13.3)
401–1000	0(0)	0 (0)
> 1000	1(0.7)	6 (4.4)

## Discussion

This study provides the first evidence from North-East Nigeria on cytomegalovirus (CMV) seroprevalence among adults living with HIV receiving antiretroviral therapy (ART). CMV exposure was universal in this cohort, with all participants demonstrating IgG seropositivity. Additionally, approximately one-third of participants (58/181; 32.1%) were IgM positive, indicating recent or reactivated infection. CMV IgM positivity was observed across socio-demographic and clinical categories; because the study was descriptive and not designed or powered to evaluate inferential associations, these distributions should not be interpreted as evidence of causal or statistical relationships.

The IgM prevalence identified here is comparable to findings from southern Nigeria: a CMV IgM prevalence of 32.7% among ART-treated HIV patients was reported in Edo State, closely matching the 32.1% observed in our cohort [8]. By contrast, a recent study from Zaria reported a substantially higher IgM prevalence of 64.1% (59/92), a difference that may reflect distinct patient mixes or methodological factors [10]. Earlier work from Ilorin similarly documented high rates of both latent and active CMV infection among people living with HIV, supporting the notion that CMV is hyperendemic across multiple Nigerian settings [7]. Systematic reviews indicate that CMV seroprevalence varies by region but is generally highest in low- and middle-income countries, frequently exceeding 90% in sub-Saharan Africa [11]. The universal IgG seropositivity observed in our study is consistent with these broader patterns [11]. 

Several factors may account for regional disparities in IgM prevalence. Differences in clinical profiles—particularly the degree of ART coverage and virologic suppression—can influence the likelihood of CMV reactivation; our cohort included a high proportion of virologically suppressed and clinically stable patients, whereas cohorts with higher IgM prevalence have included more individuals earlier in treatment or with less stable viral control [10]. Methodological differences (assay selection and interpretation thresholds) and contextual factors (population mobility, household density, socioeconomic stressors) could also modulate measured IgM prevalence between sites.

The patterns of IgM distribution across CD4 and viral load categories largely reflect the underlying characteristics of this clinic-based sample. Most participants were immunologically stable and virologically suppressed, resulting in relatively few individuals with advanced immunosuppression or high-level viremia—subgroups in which CMV reactivation and disease are classically more common. Moreover, IgM serology has known limitations as an indicator of active CMV replication: IgM may persist beyond acute infection, may cross-react with antibodies to other herpesviruses, and may not detect subclinical viral replication. Molecular assays such as quantitative CMV DNA PCR or pp65 antigenemia provide more specific measures of active replication and may reveal associations with immunologic or virologic parameters that serology does not capture.

These observations are consonant with evidence from long-term ART cohorts showing that CMV can drive chronic immune activation and features of immunosenescence even in virologically suppressed individuals. For example, Shete et al. found that higher anti-CMV antibody levels correlated with markers of immune aging among people with HIV on long-term ART [6], suggesting that CMV’s impact may manifest as persistent antigenic stimulation rather than solely as acute reactivation detectable by IgM serology.

From a public health perspective, universal CMV IgG seropositivity confirms that CMV is endemic among adults with HIV in this region and that reactivation episodes are not uncommon despite ART. However, in a predominantly stable, virologically suppressed outpatient cohort, routine universal CMV screening is unlikely to be cost-effective. A more pragmatic approach may be targeted testing and diagnostic workup for CMV in high-risk subgroups—those with advanced HIV disease, suspected treatment failure, or clinical syndromes compatible with CMV end-organ disease.

### Strengths and limitations

Strengths of this study include its status as the first systematically collected data on CMV–HIV co-infection from North-East Nigeria, concurrent measurement of CMV IgG/IgM with CD4 and HIV RNA, and a sample size adequate for descriptive epidemiology within a large tertiary ART clinic. Limitations include the cross-sectional design, which precludes temporal or causal inference; reliance on serology (particularly IgM) without molecular confirmation of active replication; and selection bias toward patients retained in care and largely virologically suppressed, which may underestimate CMV burden among those with advanced disease or poor engagement in care. The relatively small number of participants with very low CD4 counts or high viral loads also limits insight into CMV dynamics in the highest-risk subgroups.

Future research should prioritise longitudinal cohort studies that incorporate quantitative CMV DNA assays and recruit patients with advanced or poorly controlled HIV to determine whether molecular evidence of CMV replication predicts immunologic decline, virologic failure, or clinical events. Multicentre studies across different Nigerian regions would also help clarify the factors underlying regional variability in IgM prevalence and inform targeted diagnostic and therapeutic strategies.

## Conclusion

In summary, this study demonstrates that CMV exposure is universal and that recent or reactivated infection remains common among adults living with HIV on ART in North-East Nigeria. The high burden of CMV underscores the need for continued vigilance, particularly among individuals with advanced HIV disease or poor treatment response. Strengthening diagnostic capacity—especially through expanded access to CMV molecular testing—together with longitudinal research will be critical for clarifying the clinical significance of CMV reactivation and informing evidence-based strategies for integrating CMV management into HIV care programs in Nigeria and similar resource-limited settings.

## Supplementary Information

Below is the link to the electronic supplementary material.

Supplementary Material 1

Supplementary Material 2

## Data Availability

The data that support the findings of this study are available on request from the corresponding author. The data are not publicly available due to privacy or ethical restrictions.

## Abbreviations

AIDS: Acquired Immunodeficiency Syndrome
ART: Antiretroviral Therapy
CD4: Cluster of Differentiation 4 (T-cell marker)
CMV: Cytomegalovirus
DNA: Deoxyribonucleic Acid
ELISA: Enzyme-Linked Immunosorbent Assay
FTHG: Federal Teaching Hospital, Gombe
HIV: Human Immunodeficiency Virus
IgG: Immunoglobulin G
IgM: Immunoglobulin M
NHREC: National Health Research Ethics Committee
PCR: Polymerase Chain Reaction
pp65: Phosphoprotein 65 (CMV antigen marker)
PR: Prevalence Ratio
RNA: Ribonucleic Acid
SPSS: Statistical Package for Social Sciences
SSA: Sub-Saharan Africa
