# Supplementary material for: Seroprevalence of cytomegalovirus in individuals on antiretroviral therapy in a Nigerian tertiary hospital
Source: BMC Infect Dis. 2026 Mar 21;26:855. doi: 10.1186/s12879-026-13138-4 (PMC13130503; doi:10.1186/s12879-026-13138-4)
Supplement: Supplementary file 1 — Supplementary Material 1 [file 12879_2026_13138_MOESM1_ESM.docx]

**INFORMED CONSENT FORM**

**Title:** ***Cytomegalovirus Co-Infection in People with HIV on ART: Prevalence and Immunologic Correlates from a Nigerian Tertiary Hospital***

**Introduction and Purpose of Study**
You are being invited to participate in a research study that aims to determine the prevalence of cytomegalovirus (CMV) infection and its immunologic correlates among adults living with HIV who are on antiretroviral therapy. You may ask questions or seek clarification on any part of this information sheet before deciding whether to take part.

**Study Procedure**
Participation involves the collection of approximately 10 mL of blood to measure CMV antibody levels and CD4+ cell count. This procedure poses minimal risk beyond mild discomfort from venipuncture.

**Voluntary Participation and Costs**
Participation is entirely voluntary. You may withdraw from the study at any time without giving a reason, and withdrawal will not affect your medical care. There is no cost or payment associated with participation.

**Confidentiality**
All information and results obtained from you will be kept strictly confidential and used solely for research and to improve patient care. No identifying information will appear in any report or publication.

**Informed Consent**

1. I confirm that I have read and understood the information about the study.
2. I understand that participation is voluntary and that I may withdraw at any time without affecting my care.
3. I understand that authorized regulatory personnel may review my medical records for verification purposes.
4. I agree to participate in this study.

Signature / Thumbprint __________________________
Date __________________________
