# Supplementary material for: Seroprevalence of cytomegalovirus in individuals on antiretroviral therapy in a Nigerian tertiary hospital
Source: BMC Infect Dis. 2026 Mar 21;26:855. doi: 10.1186/s12879-026-13138-4 (PMC13130503; doi:10.1186/s12879-026-13138-4)
Supplement: Supplementary file 2 — Supplementary Material 2 [file 12879_2026_13138_MOESM2_ESM.docx]

**Questionnaire**

**Title: Cytomegalovirus Co-Infection in People with HIV on ART: Prevalence and Immunologic Correlates from a Nigerian Tertiary Hospital**

1. Age (years)…...........
2. Gender: Male ( ) Female ( )
3. Tribe

i.Fulani( ) ii.Hausa( ) iii.Yoruba( ) iv.Igbo( ) v.Tare( ) vi.Waja( )

vii.Tula( ) viii.Others(…………………)please specify

1. Marital status

i.Single ( ) ii.Married ( ) iii.Divorced ( ) ivWidowed ( )

5. Educational level

i.Qur,anic/Arabic only( ) ii.Primary( ) iii.Secondary( ) iv.Tertiary( ) v.None( )

6. Occupation of patient

i.Civil servant( ) ii.Housewife( ) iii.Business( ) iv.Handwork/Others( )

7. Type of family

i.Monogamous( ) ii.Polygamous( )

8. Regimen of treatment

i.First Line ( ) ii.Second Line( ) iii.Third Line( )

9. Duration on ART drugs

i.<1year( ) ii.1 – 5years( ) iii.>5years( )

10. Are you regular on your ART drugs?

i.Yes( ) ii.No( )

11. If no, why?

i.Occasional drug reactions( ) ii.Frequent drug reactions( ) iii.Others……………………………….please specify

12. Are you using traditional HIV medicine?

i. Yes( ) ii.No( )

**Laboratory Test Parameters**

CD4+ T-cell count

CMV IgG/IgM ELISA
